# B cells in human lymphoid structures

**DOI:** 10.1093/cei/uxac101

**Published:** 2022-11-12

**Authors:** Lucia Montorsi, Jacqueline H Y Siu, Jo Spencer

**Affiliations:** Peter Gorer Department of Immunobiology, School of Immunology and Microbial Sciences, King’s College London, London, UK; Peter Gorer Department of Immunobiology, School of Immunology and Microbial Sciences, King’s College London, London, UK; Peter Gorer Department of Immunobiology, School of Immunology and Microbial Sciences, King’s College London, London, UK

**Keywords:** human, B cells, lymphoid tissues

## Abstract

Most B cells in the human body are present in tissues where they support immune responses to pathogens, vaccines, autoantigens, and tumours. Despite their clear importance, they are very difficult to study and there are many areas of uncertainty that are difficult to resolve because of limited tissue access.

In this review, we consider the zonal structure of lymphoid tissues, the B cell subsets they contain, and how these are regulated. We also discuss the impact that methods of deep interrogation have made on our current knowledge base, especially with respect to studies of cells from dissociated tissues. We discuss in some detail the controversial B cells with marginal zone distribution that some consider being archived memory B cells.

We anticipate that more we understand of B cells in tissues and the niches they create, the more opportunities will be identified to harness their potential for therapeutic benefit.

## Teaser

Here, Montorsi et al consider the zonal structure of human lymphoid tissues, the B cell subsets they contain, and how zonation is regulated. The impact of that methods of deep interrogation have made on our current knowledge base, especially with respect to studies of cells from dissociated tissues is discssed. The controversial issue of B cells with marginal zone distribution that some consider to be archived memory B cells is discussed.

## Graphical Abstract

**Graphical Abstract:**
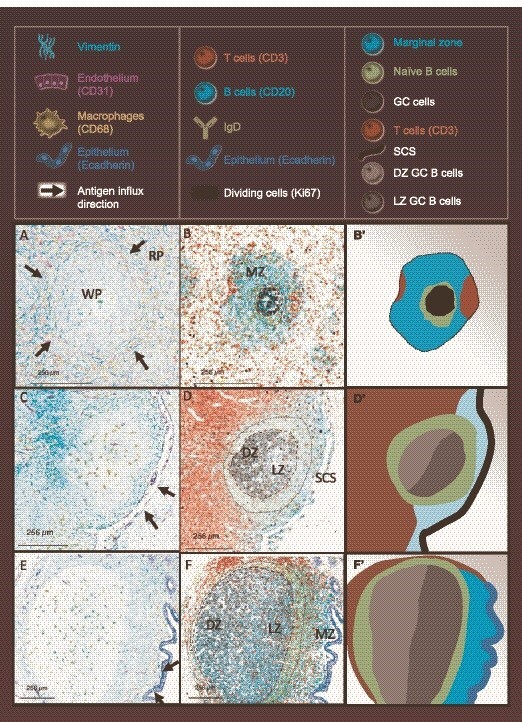

## Introduction

Lymphoid tissues are vital structures that provide reticular networks to guide immune cell recirculation and a scaffold to support the cellular interactions that are required for effective immune responses. Here we will review the features and functions of B cells in human lymphoid tissues. Plasma cells are an extensive population of B lineage cells that are diffusely infiltrated mostly within the bone marrow, intestinal lamina propria, and lymphoid tissues [1]. Because of their uniqueness, and tendency not to locate in lymphoid structures specifically, plasma cells and their immediate precursors will not be discussed here.

### B cell zones in healthy lymphoid tissues

Sub-typing B cells in cell suspensions and blood is generally dependent on either B cell surface markers, their gene expression, or their functional properties. B cells in tissues however can also be classified by their microanatomical context in tissue sections and by size, shape, and nuclear morphology [2]. This section will compare B cell zones between examples of human secondary lymphoid tissues.

Lymphoid tissue types differ markedly in their structure and function. Lymph nodes are encapsulated structures that receive lymph via afferent lymphatics that drain from the tissues into the subcapsular sinus. Afferent lymph contains dendritic cells and relatively few lymphocytes. Most recirculating lymphocytes enter lymph nodes via the high endothelial venules. Lymphocytes then exit the lymph node via the efferent lymphatics. In contrast, gut-associated lymphoid tissue (GALT) is unencapsulated. The lymphoid regions merge into the adjacent lamina propria that contains a diffuse infiltrate of immune cells, most effector cells, and connective tissue. GALT has no afferent lymphatics. Rather antigens are actively sampled by the microfold cells in the follicle-associated epithelium and delivered to the tissue below. As in lymph nodes, most lymphocytes enter via the high endothelial venules and exit via the efferent lymphatics. The spleen is a single relatively large encapsulated lymphoid organ that receives antigens via the splenic artery. Most of the spleen comprises a complex closed network of sinusoids lined with the macrophage cells that are associated with the filtration of antigens from blood. The lymphoid cells are located in the white pulp that receives antigens via the perifollicular zone of connective tissue. Lymphocytes leave the spleen in venous blood and lymphatics [3–6].

**Figure 1:**
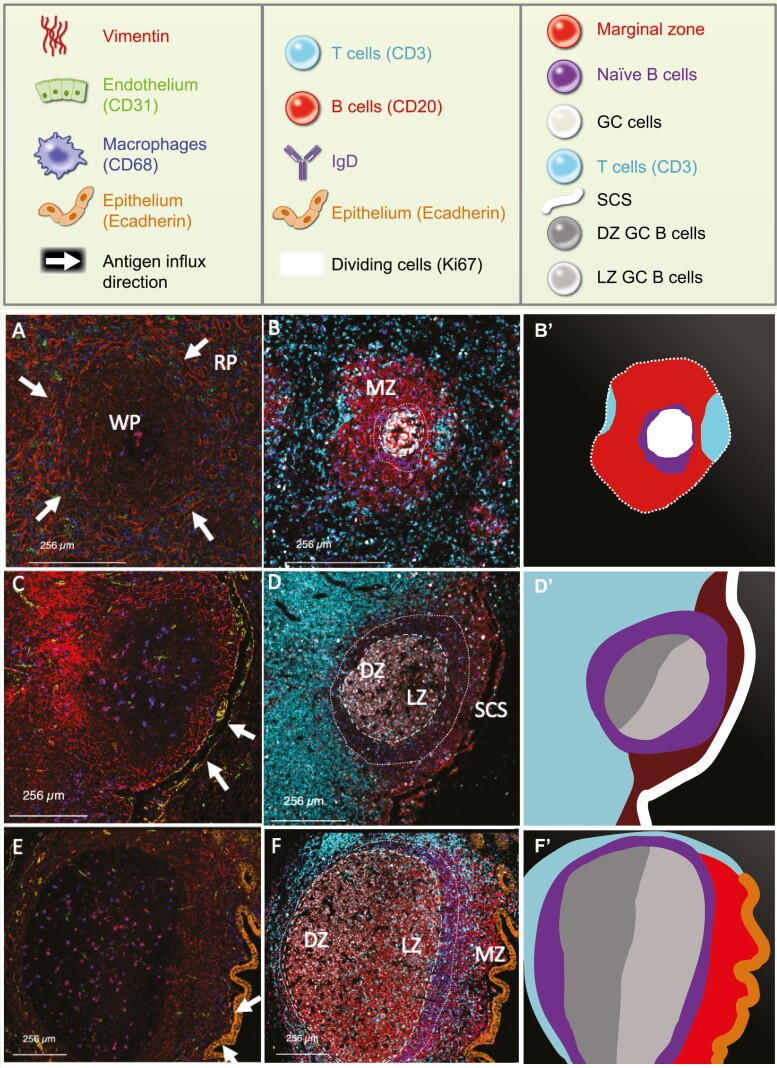
Comparison of human lymphoid tissue structures. Imaging mass cytometry representations of human spleen (A, B), mesenteric lymph node (C, D) and GALT in appendix (E,F). In A, RP: red pulp; WP: white pulp of spleen. Images in A, C, and E identify structural features of the tissues and the direction of antigen influx indicated with white arrows. Sections are coloured according to the distribution of Vimentin in red; CD31 identifying endothelium in green; CD68 identifying macrophages in blue; ECadherin that marks epithelium that is present in E and F only, in orange. Whereas lymph node and GALT are polar structures that are orientated according to the direction of antigen influx, spleen may receive antigen from around the periphery and tends to be less structurally polarized. Zones within the structures are further illustrated by tissue staining in B, D, and F, and zones identified are represented schematically in Bʹ, Dʹ, and F’. In B, D, and F, CD3 identifies T cells in cyan; CD20 identifies B cells in red; IgD identifies naïve B cells and marginal zone B cells in blue; Ki67 identifies the distribution of dividing cells in white. Zones identified by dotted boundaries are marginal zone (MZ) in B and F. The germinal centre (GC) is indicated by a dashed boundary. The GC is zonal in D and F. In the schematics in Dʹ and Fʹ, the dark zone of proliferating centroblasts is labelled DZ GC B cells and the light zone of less proliferative centrocytes is labelled LZ GC B cells. The lymph node subcapsular sinus (SCS) is identified in D and Dʹ.

The fundamental features of B cell microarchitecture in examples of secondary human lymphoid tissues are compared in Fig. 1A–F. B cell zones in lymph nodes and are strikingly polar and orientated with respect to the subcapsular sinus and the follicle-associated epithelium respectively which are known routes of antigen influx. This is most easily visualized in secondary follicles that contain germinal centres. The dark zone of proliferating centroblasts is located at the pole of the germinal centre (GC) furthest from the source of antigen. The smaller and less proliferative centrocytes are located at the opposite pole in the light zone. The mantle zone of naïve B cells that express high levels of IgM^+^IgD^+^ encircles the GC. In the lymph node, and GALT the mantle zone tends to be oval in shape with the GC located asymmetrically at one end [2, 7]. The mantle zone in the lymph node and GALT is the broadest closest to the direction of antigen influx (Fig. 1C–F). In contrast, the GC in the spleen may not retain the polar separation of centroblasts and centrocytes, and there is generally no obvious asymmetry in the mantle zone, possibly reflecting the vasculature and associated peripheral route of antigen entry, the way antigen is taken up and the systemic compartment that it serves [8–10].

In spleen and GALT the mantle zone of naïve B cells is surrounded by a clear marginal zone of B cells that are slightly larger in size than naïve B cells, have cleaved nuclei, and express low or no IgD [11]. The marginal zone of the mouse spleen contains a specialized innate-like B cell subset called marginal zone B cells (MZB cells) derived from a B cell lineage split that is dependent on B cell receptor engagement and the serine/threonine kinase Taok3 at the T1 phase [12]. The mouse splenic marginal zone also contains memory B cells [13]. Whether the human marginal zone contains both innate-like MZB cells and memory B cells is debated [14, 15]. What is not doubted is that B cells with the phenotype CD27^+^IgM^+^IgD^lo^CD1c^+^ are present in the marginal zone region of the human spleen and GALT [9–13]. The microanatomical marginal zone also contains memory B cells including class switched and IgM expressing variants that tend to occupy the most peripheral regions [12, 13, 16]. These are CD27^+^IgD^−^CD1c^−^. To avoid confusion between ‘marginal zone B cells’ (the existence of which is not universally accepted) and the marginal zone location, in this review the marginal zone B cells with CD27^+^IgM^+^IgD^+^CD1c^+^ phenotype including their circulating counterparts in blood will be referred to by the abbreviation ‘MZB cells’ [17]. The microanatomic region that contains mostly MZB cells and/or memory cells will be written in full as the marginal zone.

Cells that closely resemble MZB cells have also been described in studies of cell suspensions of tonsil and mesenteric nodes as well as in tonsil tissue sections [18] [19] [20]. However, they are not so clearly located within a distinct marginal zone region as in spleen and GALT, suggesting that the factors such as chemokines and their associated with non-haematopoietic stromal cells may guide a different microanatomic structure. In tonsil, MZB cells tend to be subepithelial [18].

The evidence for the existence of MZB cells in humans will be discussed in a later section of this review following further description of the lymphoid tissue microanatomy and the complexity of human B cell subsets.

An additional microanatomically defined zone of cells that appears specific to the spleen and that affects human B cell responses is the region of the red pulp surrounding the outermost margins of the white pulp. Neutrophils that can provide help for B cells occupy this area. Mechanisms involve the production of BAFF, APRIL, pentraxin 3, and IL-21 [21, 22].

Microanatomical variability in B cell function may be locally inducible. B cells in close proximity to epithelium, where epithelium is present on the margins of lymphoid tissue for example tonsil or GALT, may express the Fc receptor-like glycoprotein FcRL4 [19, 23]. The encoding gene was identified at a chromosomal breakpoint in the case of marginal zone B cell lymphoma of mucosa-associated lymphoid tissue (MALT lymphoma). There is no mouse homologue. Despite being expressed predominantly by CD27^-^ cells, FcRL4 has been associated with memory B cells in a number of studies [24–27], though the cells that express it seem to be phenotypically heterogenous [28]. It is possible that FcRL4 is induced locally by epithelium and that it is not associated with a B cell subset *per se* [19]. Functionally it is an inhibitory receptor that binds aggregated monomeric IgA. Although it dampens B cell responses initiated through the B cell receptor [29, 30], it enhances responses to TLR9 activation suggesting it could move B cells from adaptive to more innate-like function [31]. Being associated with epithelial surfaces, B cells expressing FcRL4 are rarely seen in blood, except in some infectious diseases including HIV and malaria [32–34].

### Regulation of human lymphoid tissue microanatomy

The formation of lymphoid structures in mice is dependent on lymphoid tissue inducer cells (LTi) that express the RORγt transcription factor and that require IL-7 for their development. They interact with and modify stromal cells via lymphotoxin [35, 36] resulting in the recruitment of leukocytes. In humans, this appears to be broadly true. However, analysis of lymphoid tissues in individuals with deficiencies in IL7R has identified that LTi-independent mechanisms may also exist that orchestrate formation of lymphoid structures that drain the gut [37].

Microanatomical zonation within tissues appears conserved between species and is regulated largely by chemokines and their receptors. Recruitment to the T cell zone is dependent on the interactions between CCR7 that is expressed by most lymphocytes, and its ligands the chemokines CCL19 and CCL21 that are secreted by stromal cells such as the fibroblastic reticular cells within tissues. CCL21 is also expressed by high endothelial venules (HEV) and plays a role in the recruitment of leukocytes from blood across HEV and into the tissues [38–42]. CCL19 from stroma can be transcytosed into HEV and can also contribute to lymphocyte extravasation. CCL21 has a C terminal tail that facilitates efficient binding to the extracellular matrix via glycosaminoglycans and it can therefore mediate traffic under shear forces. CCL19 in contrast tends to remain soluble and thus supports migration but not adhesion [42].

The B cell zone is shaped in part by the expression of CXCR5 by B cells and also by recently activated T follicular helper cells (Tfh) [43]. Migration and coalescence of CXCR5 expressing cells allowing them to form the B cell follicle is guided by CXCL13 produced by cells in the GC including the specialized stromal cells termed the follicular dendritic cells (FDC) and also by GC located Tfh in humans [44–46]. The secreted CXCL13 appears predominantly to form gradients that are matrix bound via heparin or heparan sulphate in humans. Such bound CXCL13 can be released by cathepsin B. An elegant combination of observational science and computational analysis of tonsil tissue demonstrated complexity in CXCL13 form and location that is likely to guide human B cell subsets between different functional areas of the B cell zone [47, 48].

The polarized structure of the GC itself is also chemokine regulated. Dividing centroblasts in the dark zone of the GC express the chemokine receptor CXCR4 enabling them to migrate towards CXCL12 produced by dark zone stromal cells [49, 50]. Centrocytes express higher levels of CXCR5, supporting migration towards CXCL13 produced in the light zone [49]. Movement between the light and dark zone appears to be regulated in part by interactions between innate lectin-like transcript 1 (LLT1) on B cells and CD161 on FDCs via CD83 upregulation and CXCR4 downregulation [51]. Exit from the GC as memory B cells associated with expression of CCR6 in humans [52]. CCR6 makes other contributions to the structure of lymphoid tissue including regulation of B cell dynamics around the subepithelial dome and GC in mouse models [53]. The human mucosal epithelium is a source of CCR6 ligand CCL20 though whether this is reflected in human mucosal B cell traffic or function or the shaping of mucosal lymphoid tissue architecture is as yet unknown [54].

This functional dynamic of GC activity in human lymph nodes and generation of affinity matured B cell responses has recently visualized in the immune response to BNT162b2, an mRNA-based vaccine that encodes the full-length SARS-CoV-2 spike (*S*) gene. The generation of persistent GC responses was demonstrated. GC cells and neutralizing antibodies alongside memory B cells and plasmablasts were sustained for at least 6 months [55, 56].

### B cells studied in dissociated human tissues

Images of B cells in tissue sections can be considered to be a single frame time point in a movie of immune physiology and active response dynamics. They can identify cellular interactions in space at a moment in time and provide a representation of the relative frequencies and migratory tracks of broadly defined B cell types. However, visualizing phenotype and transcriptome with the highest resolution currently requires analysis of cell suspensions derived from tissues [57].

#### Diversity of pre-GC B cells in human tissues

Whilst most available information relates to the diversity of previously activated B cells, diversity in transitional and naïve B cells also exists. Maturation of the naïve repertoire involves positive and negative selection events, though how this impacts cell phenotype and tissue anatomy is not yet known [58]. Studies of naïve and transitional cells in blood identified groups of B cells from the T2 stage that differed in their level of expression of IgM, IL4R and α4β7 integrin that mediates movement into mucosal tissues via recognition of endothelial MAdCAM-1 or that differ in purine metabolism [57, 59, 60]. Whilst IgM^lo^ T2/3 cells were identified as putative precursors of follicular naïve B cells, the IgM^hi^ T2 cells have been linked to a pathway towards the development of MZB cells that are also IgM^hi^ and that undergo a maturation phase in the GC. GALT is chronically stimulated and has GC from shortly after birth and therefore provides a life-long niche to support this activity. Early data on lymphocyte traffic in sheep, rats, and mice suggested that naïve B cells migrate randomly and that differential migration between systemic and mucosal sites occurs only after imprinting associated with antigen encounter [61, 62]. However, MZB cell development varies considerably between species and there is no current animal model for the developmental trajectory observed in humans.

It has been proposed that a subset of B cells in the tonsil express CD5 and that they may be analogous to the innate-like B1 subset in mice [63, 64]. However, this has not gained general support. B cells in human fetal tissues express CD5, as do a subset of B cells beneath the tonsillar epithelium, and also transitional B cells [18, 65]. Most cases of the B cell lymphomas of small lymphocytic lymphoma and mantle zone lymphoma appear to be derived from immature B cells that also express CD5 [66]. A functional study of the umbilical cord and blood B cells identified cells with the phenotype CD20^+^CD27^+^CD43^+^CD70^−^ that had properties associated with the murine B1 cells such as the production of natural antibodies, rapid response to activation, and tonic signalling [67]. A similar subset has now been identified in a deep multi-organ analysis of human fetal B cell subtypes. In addition to the expression of CD20^+^CD27^+^CD43^+^ and CCR10, a high proliferation fraction compared to mature B cells, and greater secretion of antibodies were observed. The B1 B cells described had shorter rearranged immunoglobulin heavy and light chain gene CDR3, due to lower addition of N and P nucleotides [68]. Innate-like B cells that have been compared to B1 cells have been observed in human renal allografts [69]. This interesting field is still developing and the benign analogues of CD5^+^ B cell lymphomas and the existence of human B1 cells are likely to gain resolution in the near future.

#### Diversity of post GC B cells in human tissues

Considerable diversity in memory B cells within and between tissues has been described. The expression of CD27 has been a valuable marker of human memory B cells, though CD27-memory B cells exist. On the one hand, CD27^+^ and CD27^−^ variants within single clones of B cells in blood have been described suggesting that these can be developmental states within a clone [70]. On the other hand, it has been proposed that these are functionally different and that they derive from T cell dependent and T cell independent responses respectively [71]. CD27 expression variants exist. CD27^bright^ and CD27^dull^ subsets have been described in blood and spleen that progress developmentally from dull to bright stages and become enriched in the more mature bright phase during pregnancy, resolving back to the more ‘flexible’ CD27^dull^ stage post-partum [72].

The glycosylated form of the CD45RB leukocyte common splice variant recognized by the monoclonal antibody MEM55 has been of great value in resolving subsets of memory B cells in tissues. This CD45RB^MEM55^ variant was first described by the Mats Bemark group as a marker of memory B cells including the CD27^-^ memory variants, and thus promised to be a better marker of memory than CD27 itself [73, 74]. The same group subsequently identified that this antigen was also expressed by an IgM^hi^ subsets of more immature cells and this has also been key to the identification of MZB cell differentiation through its expression by CD27^−^ MZB precursors [75, 76].

Memory B cell subsets that are represented with different frequencies between tissues have been described. Memory B cells expressing CD45RB include a gut resident subset also expressing CD69. In this case, gut residency was determined by the identification of this phenotype in the B cell preparations from the gut predominantly but not blood or bone marrow, and also the previous association made between the expression of CD69 *per se* and tissue residency [77]. Weisel *et al*. identified that CD74 and CD370 were more highly expressed by memory B cells from the spleen compared to memory B cells from other sites [78].

Phenotypic markers have been identified that are upregulated in memory compared naïve B cells including CD79b, CD1c, CD48, CD298, CD29, CD24, CD45RB, CD27, CD97, CD206, CD183, CD43, CD63, CD54, CD84, and CD81 [78, 79]. Expression of CD11a and CD200 alongside CD45RB^MEM55^ is a key combination for the identification of memory B cells [78]. In addition, based on complex phenotyping, Glass *et al*. propose that CD27^-^, CD45RB^+^ cells are early memory; that a tonsil-resident CD39^+^ population predominantly identifies class switched memory and that a CD19^hi^CD11c^+^ memory population exists that is highly responsive to activation [79]. Deep phenotyping studies of highly complex datasets are likely to be important reference points going forwards. At the moment, many key findings are difficult to resolve in terms of precise relevance to regional immune responses and immune-mediated pathologies.

Activated memory B cells (ABC) are a Tbet expressing tissue-resident subset that accumulates with age. Tbet expression by ABC may be high or low [80]. In humans, Tbet^hi^ cells can be identified in blood and tend to reside in the spleen and bone marrow but are not found in the lymph node, tonsil, or lymph, and do not therefore recirculate via conventional nodal trafficking routes. Tbet^lo^ cells in contrast can be identified in tonsil, lymph nodes, and lymph. Specificity for influenza HA antigens could be observed amongst Tbet^hi^ cells in spleen but not lymph nodes and they are therefore likely to be significant contributors to the splenic influenza specific response. However, this was found to be highly variable between individuals, and the significance of Tbet^hi^ and Tbet^lo^ variants within the human B cell response and the relationship between Tbet^hi^ and Tbet^lo^ cells remains unclear [80, 81]. An additional subgroup of Tbet expressing B cells referred to as ‘atypical B cells’, that lack CD27 and CD21 increase in the blood in malaria infection and that can differentiate rapidly into antibody-secreting cells with help from Tfh [82]. How the Tbet expressing B cell subsets relate to each other is as yet uncertain.

### Marginal zone: a reservoir of memory cells or a zone that includes innate-like B cells?

**Figure 2.**
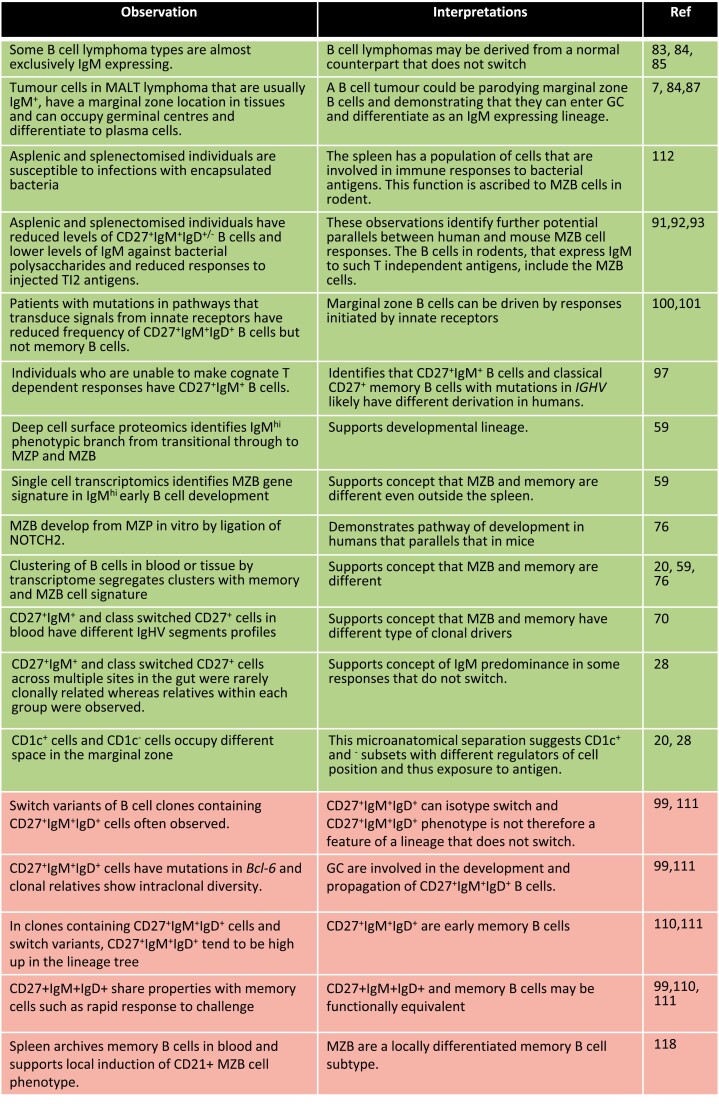
The relationship between MZB cells and unswitched memory B cells is unclear and debated. Dissection of the case for and against the existence of marginal zone B cells as a group of cells that is separate from conventional memory. Observations and associated interpretations for the existence of MZB cells as a distinct entity are on a green background at the top of the chart. Evidence that cells with the phenotype CD27^+^IgM^+^IgD^+/−^ are unswitched memory cells is on a pink background in the bottom 5 rows of the chart.

Memory B cells can be divided into subsets according to Ig isotype expression. The relevance of IgM memory B cells and of co-expression or not of IgD by CD27^+^ B cells in tissues is highly controversial and we will discuss this here. The issues are summarized in Fig. 2. B cells expressing other Ig isotypes will be reviewed comprehensively elsewhere in this issue.

#### The case for an innate like, IgM-expressing MZB cell type in humans

The concept of human B cells expressing exclusively IgM with lack of progression to class-switched variants, despite showing hallmarks of prior activation evolved from an observation that some histogenetic types of human B cell lymphomas had somatically mutated immunoglobulin variable region genes (IGV) indicative of GC derivation, but tended to express IgM and not undergo class switching [83]. Such tumours rarely exist expressing isotypes other than IgM. Marginal zone B cell lymphoma of mucosa associated lymphoid tissue (MALT lymphoma) is one such malignancy that could be observed histologically to infiltrate GCs and undergo plasma cell maturation as IgM expressing cells [84, 85], and to have somatic mutations in IGHV [86]. If MALT lymphomas express IgD at all, this is at a low level.

When considering potential benign analogues of the malignant B cells in MALT lymphoma, a resemblance between tumour cells and normal splenic and mucosal marginal zone B cells in multiple parameters was noted. These similarities included nuclear morphology, cell size, cell phenotype including expression of IgM and a tendency for tumour cells to encircle the zone of naïve B cells [85, 87]. The resemblance drove the marginal zone B cell classification of MALT lymphomas [88].

It was subsequently shown that B cells in the normal human marginal zone isolated by microdissection from tissue sections are GC-experienced cells because they have somatic mutations in their IGV genes [89]. This contrasts with B cells in the rodent marginal zone that are static naïve cells [12, 90]. However, this is consistent with the alignment with MALT lymphoma as a MZB cell type since it also had mutated IGV, and the majority express IgM [83, 86].

Functional aspects of the splenic B cells ascribed in rodents to MZB such as an ability to mount B cell responses to T independent type 2 antigen appeared conserved across species [91–93]. Associated with this, splenectomized individuals or individuals with asplenia, who often suffer from infections with encapsulated bacteria, have reduced frequencies of CD27^+^IgM^+^IgD^+^ B cells. Likewise, the development of the marginal zone over the first 2 years of life and the appearance of CD27^+^IgM^+^IgD^+^ B cells in blood also occurs over the first 2 years of life and parallels the presence of anti-polysaccharide IgM in serum [94–96].

An interesting series of papers further analysed circulating CD27^+^IgM^+^IgD^+^ cells [17] that were also present in cases of CD40L deficiency when IgM-only cells were absent [97]. Since patients with CD40L deficiency have reduced GC formation due to the lack of cognate interaction between B and T cells, these authors proposed that CD27^+^IgM^+^IgD^+^ cells are associated with T-cell-independent B cell responses; a feature associated with marginal zone B cell function [97]. They showed that these cells shared features with innate-like B cells in the marginal zone and proposed that CD27^+^IgM^+^IgD^+^ cells are circulating MZB counterparts [17]. A study of sorted CD27^+^IgM^+^IgD^+^ and GC cells from human GALT, using bulk sequencing of IGHV genes, identified clones with members in both compartments, thus demonstrating that CD27^+^IgM^+^IgD^+^ can acquire mutations in IGV in the GC of GALT [28]. Lymphoid tissues in CD40L deficiency can contain small abortive GC that may support somatic hypermutation and that could be responsible for the low level of observed mutations in IGHV in this patient group [98]. CD27^+^IgM^+^IgD^+^ cells that tended to have mutations in IGV also had mutations in *Bcl6* that are transcribed during the GC response confirming that they had undergone a GC response [99]. Interestingly, MZB cell depletion without depletion of memory B cells has been observed in patients with immunodeficiencies affecting innate immune signalling [100, 101]. These patients tend to suffer from recurrent bacterial infections thus providing compelling evidence that innate-like CD27^+^IgM^+^IgD^+^ B cells are functionally distinct and important for the maintenance of good health.

Further evidence for MZB as a state along a developmental pathway separate to IgM memory, is derived from the observed maturation of splenic CD27^+^IgM^+^IgD^+^ cells from CD27^-^CD45RB^hi^ MZB precursor cells (MZP) [76]. This is dependent on ligation of Notch2 by DLL1 that is expressed through the stroma of the splenic marginal zone, and has also been observed in the subepithelial dome of GALT [102]. A developmental trajectory of B cells that express high levels of IgM can be observed by deep phenotypic analysis and by single-cell transcriptomics. The developmental branch starts at the T2 stage and progresses via IgM^hi^ naïve B cells through to MZP before maturing into MZB cells. B cells on this developmental axis in blood express relatively high levels of β7 integrin that mediates cellular extravasation into GALT. This is consistent with the role of GALT GC in MZB development [59]. Undirected clustering analysis of B cells according from blood and tissues according to the transcripts of single cells consistently separates memory B cells expressing HOPX and COCH from MZB cells that express CD1c and PLD4 for example [59, 76].

MZB cells can be identified histologically using multiplexed tissue staining methods. They tend to be located around the periphery of the mantle zone of naïve B cells. MZB cells in turn tend to be surrounded by memory B cells that express neither IgD nor CD1c [17, 20].

**Figure 3.**
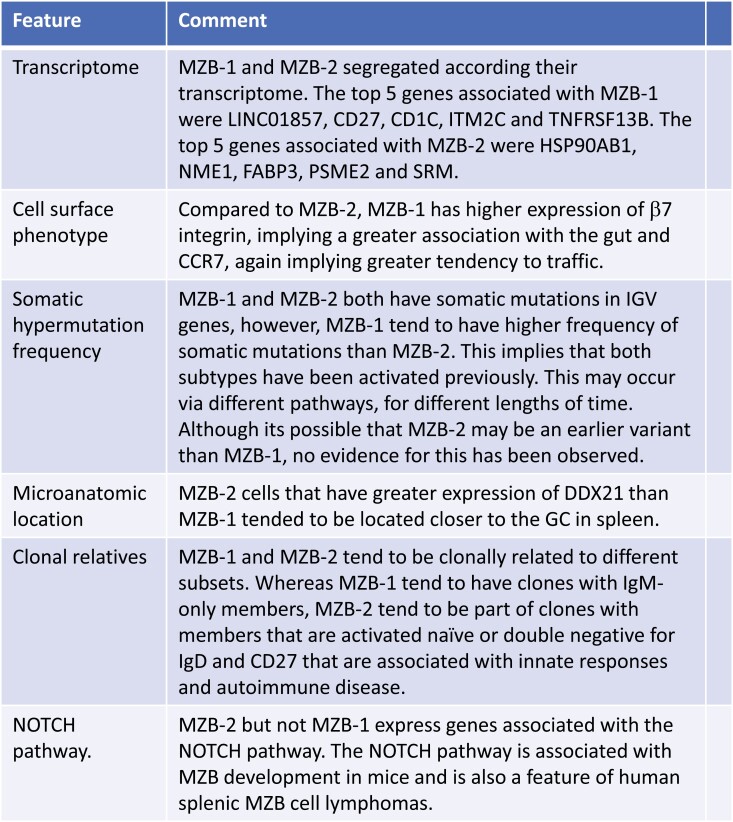
Heterogeneity in human MZB-cells. Features of groups of MZB cells identified by deep proteomics and transcriptomics in ref. 20.

Repertoire studies do not support the view that CD27^+^IgM^+^IgD^+^ and CD27^+^IgM^+^IgD^-^ populations are the same since they have different usage of IGHV gene segments. Most notably IGHV1-18, IGHV1-2, IGHV1-46, IGHV1-69, and increased IGHV3-23 in IgM memory compared with switched memory [103]. In addition, CD27^+^IgM^+^IgD^+^ has a strong tendency not to undergo class switch recombination compared to IgM-only cells in GALT [28]. It is known that AID catalysis of cytidine to uracil is essential for both somatic hypermutation and class switch recombination. However, the mechanism is different and they do not necessarily occur simultaneously after B cell activation, therefore hypermutation without switching is mechanistically feasible [104, 105]. Heterogeneity in CD1C^+^ MZB cells has been described according to differences in phenotype and transcriptome. Variants differ in their homing profiles, their location within the marginal zone, their link to the Notch pathway, and their closest clonal relatives as summarized in Fig. 3 [20].

#### Could all marginal zone B cells be memory cells?

A seminal paper from Klein, Kuppers, and Rajewsky, 1996 introduced the concept of IgM memory B cells in humans with mutated IGV genes [83]. They used the term ‘IgM only’ cells in this context for the cells that expressed IgM but not IgD. Many studies refer to cells with either the phenotype CD27^+^IgM^+^IgD^+^ or CD27^+^IgM^+^IgD^-^ as unswitched memory B cells [106–109]. In common with switched memory B cells, the majority of CD27^+^IgM^+^IgD^+^ and CD27^+^IgM^+^IgD^-^ cells have the hallmarks of having emerged from GC responses in their somatically mutated IGV genes [99]. Moreover, memory B cells can certainly occupy the marginal zone and therefore there is no ‘need’ to infer a separate MZB cell type that has this property. Work including the functional comparison of CD27^+^IgM^+^IgD^+^ cells with CD27^+^IgM^+^IgD^−^ cells concluded that they are not different [110] [111]. Many (though not all) studies have identified class switch variants of both CD27^+^IgM^+^IgD^+^ and CD27^+^IgM^+^IgD^−^ cells and propose that IgM clone members present at the same timepoint as the switched variants represent cells that branched away early in the same maturation process [28] [103] [111]. Consistent with this, IgM only and CD27^+^IgM^+^IgD^+^ cells have lower mutational frequencies than switched memory cells and CD27^+^IgM^+^IgD^+^ cells have the lowest [28]. It was shown that CD27^+^IgM^+^IgD^+^ are transcriptionally similar to IgG memory cells and share traits of memory B cells such as more vigorous restimulation potential. CD27^+^IgM^+^IgD^+^ cells showed a high potential to be stimulated by activated neutrophils and were also enriched in IFN-gamma receptor 1 (IFNGR1) transcripts and related function compared to IgG memory or naive B cells. In terms of lineage tree analysis CD27^+^IgM^+^IgD^+^ cells were observed to be early variants within clones containing class-switched variants [110, 111].

A subset of both IgM and class-switched CD27^+^ B cells in the spleen can express high levels of CD21 and low levels of CD23 compared to other B cells. It has been proposed that memory B cells can be recruited into the spleen as an archived sample of memory over time and that CD21 can be locally induced by stromal DLL1 so that individuals acquire a CD21^hi^ marginal zone subtype through life. In this case, the definition of MZB hinges on the expression of complement receptors acquired during the archiving of memory B cells into the marginal zone [99].

As well as the undoubted importance of IgM responses initiated in the spleen [112] IgM responses have a specialized role in mucosal protection alongside IgA [113]. IgM memory cells have been described in GALT that are clonally related to lamina propria IgM plasma cells. This study also described that the IgM memory cells in the gut had different transcriptional profiles to splenic marginal zone B cells [113].

### Functions of B cells in tissues other than Ig secretion

B cells are potent antigen presenting cells that have roles in, for example, selection during affinity maturation by presenting antigen to Tfh, in thymic selection, and in the maintenance of T cell memory, which almost by definition must occur in tissues [114, 115]. Antigen presentation by B cells has been shown to be major driver of the T cell response to gluten peptides potentially cross-linked to tissue transglutaminase in coeliac disease [116]. This model of B cells driving subsets of pathogenic T cells could be relevant to other autoimmune diseases that include pathogenic T cells, but that respond well to B cell depletion, such as rheumatoid arthritis.

The sampling of dendritic cell surfaces by B cells in tissues by trogocytosis has been observed [117]. In mice, this was attributed to marginal zone B cells that acquired membrane via B cell complement receptor binding to C3 that was covalently linked to dendritic class II MHC. Human class II can also bind C3 [117] and marginal zone B cells in humans express high levels of complement receptor CD21 [118] suggesting that this pathway may be involved in the co-operation between B cell and dendritic cells and the acquisition of novel functional properties.

B cells are known to produce cytokines and can be potent regulators of immune responses. This important B cell function will be described in another review in this series.

### Tertiary lymphoid structures

Whilst most lymphoid tissues are constitutive and present (albeit inactive) before birth, lymphoid tissues can also be acquired *de novo* in response to infection, during autoimmune responses, and in cancer.

A well-described example of the acquisition of lymphoid tissue in response to infection is the mucosa-associated lymphoid tissue acquired in the stomach in response to infection with *Helicobacter pylori* [119]. Whilst a normal stomach is devoid of lymphoid tissues and protected by innate mechanisms such as gastric acid, *H. pylori* can neutralize the acidic environment by secreting urease that generates ammonia from urea. The presence of structured lymphoid tissues in the stomach acquired in response resembles normal GALT and the presence of acquired GALT has a very strong positive association with *H. pylori* infection.

Lymphoid tissue can be acquired at sites of pathology in autoimmune diseases, such as in the inflamed synovium in rheumatoid arthritis. The appearance can range from diffuse infiltrates of mixed leukocytes, through to organised lymphoid structures with active GC responses that support AID mediated diversification of the antibody repertoire and the generation of autoimmune specificities [120–122].

**Figure 4.**
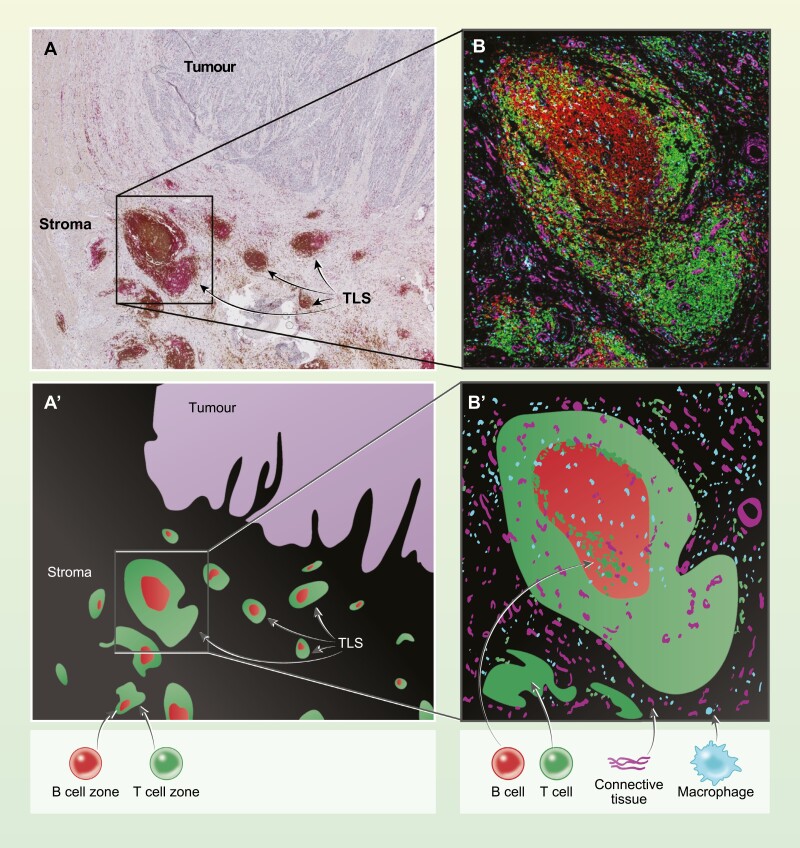
Tertiary lymphoid structures in colorectal cancer. Illustration of tertiary lymphoid structures (TLS) in colorectal cancer. In A, B cells are stained using an antibody to CD20 (brown) and T cells using an antibody to CD3 (pink). TLS tends to be located in the stromal tissue, often on the boundary of the tumour in colorectal cancer. The tumour is indicated with a black arrow in A. In B, imaging mass cytometry is used to visualize a TLS in a serial section to that illustrated in A. In B, B cells are identified by CD20 in red, T cells by CD3 in green, macrophages by CD68 in cyan, and vimentin expressed by connective tissue in magenta. The regions identified by staining in A and B are represented schematically in Aʹ and Bʹ respectively.

Although tumours may include B cells in stromal compartment, these are most often found in structurally organized tertiary lymphoid structures (TLS). Like ectopic follicles found in autoimmune syndromes, TLS can be simply organized in separate B and T cells zones or include a complete GC in their most mature form (Fig. 4). The presence of TLS, especially mature ones, has been widely associated with better prognosis and response to therapies, including checkpoint inhibitors, in various solid tumours [123]. While recent reports identify TLS as sites of B cell maturation, and of generation of anti-tumour antibodies [124], others highlight the importance of other cells types in their anti-tumour activity, particularly of B cell follicle associated T cells and DC [125, 126]. Because of its importance, for example to the understanding mechanisms of action of therapeutics, this is an area of high current interest.

### B cells in tissues: a future outlook

Human tissue research has benefitted massively from the development of novel systems such as *in vitro* assembled organoids that allow direct investigation of antigen-specific responses [127], advanced analysis of B cell receptors in a translational context [56], and from methods for deep and high-resolution analysis of transcriptomes and cell surface phenotypes from multiple human tissues [128, 129]. Computational methods for analysis are also developing in parallel and are becoming more accessible to the scientific community in general. We anticipate that the next leap forwards in understanding the physiological relevance of tissue architecture and tissue-based cellular interactions and events in humans will be provided by increasing the resolution of spatial transcriptomics that will match microanatomy with transcriptome and B cell clones defined by IGV gene sequence. Most human immune-mediated pathology manifests in tissues and understanding the detail and complexity is likely to provide important future advances in translational immunology.

## Data Availability

This is a review document that does not contain original data.

## Abbreviations

ABC: activated memory B cells
FcRL4: Fc receptor-like glycoprotein 4
FDC: follicular dendritic cells
GALT: gut-associated lymphoid tissue
GC: germinal centre
HEV: high endothelial venules
IFNGR1: IFN-gamma receptor 1
IGV: immunoglobulin variable region genes
LLT1: innate lectin-like transcript 1
LTi: lymphoid tissue inducer cells
MALT lymphoma: marginal zone B cell lymphoma of mucosa-associated lymphoid tissue
MZB: marginal zone B
MZP: MZB precursor cells
S: SARS-CoV-2 spike
Tfh: T follicular helper cells
TLS: tertiary lymphoid structures
